# The novel angiogenesis regulator circFAM169A promotes the metastasis of colorectal cancer through the angiopoietin-2 signaling axis

**DOI:** 10.18632/aging.204974

**Published:** 2023-08-23

**Authors:** Zhiwei Wu, Fan Zhang, Shaobin Huang, Ming Luo, Kai Yang

**Affiliations:** 1 Department of General Surgery, Changsha Central Hospital affiliated to University of South China, Changsha, Hunan 410000, China; 2 Department of General Surgery, the Third Xiangya Hospital of Central South University, Changsha, Hunan 410000, China; 3 Department of Organ Transplantation Center, Xiangya Hospital, Central South University, Changsha, Hunan 410000, China

**Keywords:** angiogenesis, colorectal cancer, ANGPT2, circFAM169A, miR-518a-5p

## Abstract

Background: Angiogenesis plays an important role in the metastasis of cancers. However, the mechanisms whereby circular RNAs (circRNAs) regulate angiogenesis and affect cancer metastasis are still unclear.

Methods: We used gene set variation and Spearman’s correlation analyses to identify novel angiogenesis-related circRNAs, including circFAM169A. The Kyoto Encyclopedia of Genes and Genomes and Gene Ontology were used to assess the potential biological function of circFAM169A. A quantitative reverse transcription–PCR (qRT-PCR) analysis of 20 pairs of colorectal cancer (CRC) samples was performed to detect the expression level of circFAM169A. Transwell assays, tube formation assays, and nude mouse metastatic tumor models were used to study the function of circFAM169A in CRC. qRT-PCR, dual-luciferase reporter gene assay, RNA antisense purification assay, and Western blot were performed to analyze the competing endogenous RNA mechanism of circFAM169A in promoting CRC angiogenesis.

Results: circFAM169A was highly correlated with the hallmark of angiogenesis in CRC patients. It was up-regulated in liver metastasized CRC patients. circFAM169A overexpression promoted the angiogenesis, migration, and invasion of CRC cells while its down-regulation had the opposite effects. *In vivo* mouse models further highlighted the pro-metastatic role of circFAM169A in CRC. More importantly, we discovered that circFAM169A enhances the expression of angiopoietin-2 by binding to miR-518a-5p.

## INTRODUCTION

Colorectal cancer (CRC) is the fourth most deadly malignancy in the world. Metastasis contributes heavily to CRC-related mortality [1], and the treatment of metastatic CRC remains a huge challenge. Metastasized cancers, in general, rely on neovascularization for adequate oxygen and nutrition despite some of the drawbacks of this process, such as immature vascular structure, twists, poor permeability, and dysfunction [2]. Angiogenesis, a complicated multistep process, is a prerequisite for CRC metastasis [3]: disrupting angiogenesis can effectively inhibit cancer metastasis. Anti-angiogenic drugs have always been the focal point in the development of anticarcinogens [4, 5]. However, the mechanisms of cancer angiogenesis are still poorly understood, and new angiogenic factors in CRC must be intensively studied to uncover new potential anticancer targets bearing clinical implications for the diagnosis and treatment of CRC.

Circular RNAs (circRNAs) – closed-loop noncoding RNAs lacking a 5′cap and a poly (A) tail [6] – can regulate tumor progression through multiple mechanisms acting at the epigenetic, transcriptional, and post-transcriptional levels [7]. Of them, the sequestration of microRNAs (miRNAs) is emerging as a widespread cancer-regulating mechanism. For example, circCCT3 regulates the expression of vascular endothelial growth factor A (VEGFA) and WNT by sponging mir-613 [8], while circ_0056618 promotes the expression of C-X-C chemokine receptor type 4 and VEGFA by sponging mir-206 [9], thus stimulating CRC angiogenesis. Presently, cancer angiogenesis research is mainly focused on VEGF, but some studies have identified that circRNA can regulate cancer angiogenesis via AGGF1, ZIC4, and SOX13 [10, 11]. circFAM169A, also known as circ0007158 or circRNA 103890ic, is a circular RNA that is formed from exons 1–7 of its precursor FAM169A pre-mRNA on chromosome 5. A previous study has identified that Circ0007158 could regulate intervertebral disc degeneration by targeting miR-583 and BTRC [12]. Another study has showed that Circ0007158 is potentially associated with the overall survival of CRC patients [13]. However, no previous study has explored its function in cancers. Angiopoietin 2 (ANGPT2) is a well-known growth factor involved in angiogenesis [14], but its potential function in CRC has never been investigated.

In this study, by creatively screening circRNAs that may activate angiogenesis using gene set variation analysis (GSVA) and other bioinformatic analyses, we identified circFAM169A as a candidate for further mechanistic investigation. We determined its expression level in clinical CRC samples, validated its differential expression in our own clinical sample-based RNA-seq data, and further defined its ability to affect angiogenesis. Importantly, we explored its possible regulatory mechanism, focusing on a competing endogenous RNA (ceRNA) network. We revealed that circFAM169A regulates CRC angiogenesis via the circFAM169A –miR518a–ANGPT2 axis.

## METHODS

### General data information

Datasets GSE147711 is the expression data for 20 patients with colorectal cancer which based on Arraystar Human CircRNA microarray and Exiqon miRCURY LNA microRNA array. It was downloaded from Gene Expression Omnibus (GEO) database (https://www.ncbi.nlm.nih.gov/geo/). We obtained the circRNA, miRNA and mRNA expression data from 20 CRC patients with or without liver metastasis.

### Gene set variation enrichment and correlation analysis

Gene set variation analysis (GSVA) were used to estimate the relative enrichment of a gene set of interest over a sample population, which is used to observe the variation in the activity of a set of genes corresponding to a particular biological process [15]. Followed by previous study [16], the GSVA R package [17] was used to analyze the pathway between two different groups of samples, and the defined gene set: HALLMARKS was downloaded from the Molecular Signature Database. “zscore” algorithm was used to give score to each sample and used this enrichment score to represent the degree of absolute enrichment of a gene set hallmark, while different scores also represent the activity degree of the hallmarks in the sample. Then we calculated the correlation of each circRNA and hallmark scores by calculating the Person correlation coefficients, which was also calculated by R software.

### Differentially expressed circRNA analysis

The DEGseq2 package was used to identify the differentially expressed circRNAs between CRC patients with/without liver metastasis from GSE147711. After data normalization, all the analyses were performed using the cutoff criterion: *P* < 0.05 and |Log2FC| > 1.

### Gene enrichment analysis

#### Gene ontology and Kyoto Encyclopedia of Genes and Genomes enrichment analyses

To explore the circRNA at functional levels, Gene Ontology (GO) and Kyoto Encyclopedia of Genes and Genomes (KEGG) pathway functional enrichment analyses were performed. GO analysis includes the categories of molecular function (MF), cellular component (CC), and biological processes (BPs). Pathway analysis is the process of classifying large genes by the KEGG database. In this study, the high-level group and low-level group were determined based on the expression level of the circRNA. Differential expression genes between two groups were identified using DESeq2 R package [18]. GO terms with *P* < 0.05 were considered significantly enriched. In KEGG, and pathways with a Fisher’s exact test *P* < 0.05 were considered significantly enriched.

### Protein-protein interaction network

Search Tool for the Retrieval of Interacting Genes/Proteins (STRING) (https://string-db.org/cgi/input.pl) is an online database search tool for the retrieval of interacting protein [19]. The DEG were used to determine the potential functional protein network that might be affected by the specific circRNA. In this study, the cytoscape software (version 3.7.2) was used to construct a PPI network based on the data retrieved from STRING.

### Tissue specimens

Cancer and paracancerous tissues were collected from 20 patients with CRC who were pathologically diagnosed from January 1, 2018, to December 31, 2022, at Department of General Surgery, Third Xiangya Hospital, Central South University. Patient inclusion criteria are as follows: (1) The patients were diagnosed with CRC and admitted to our hospital for surgical treatment. (2) The patients had not been treated before surgery. (3) The patients had complete pathological and clinical data.

### Cell culture

HCT116 cells, RKO cells, SW480 cells, HCT8 cells, HT29 cells, LOVO cells, and FHC cells were obtained from the KeyGEN BioTECH (Nanjing, Jiangsu, China). Cell culture was performed in an atmosphere of 5% CO_2_ at 37°C.

### Plasmid, lentivirus and siRNA construction

The ANGPT2 overexpression, the circFAM169A overexpression and interference lentiviruses used in this study were purchased from Tsingke Biotech (China). The ANGPT2-siRNA, the mimics and inhibitor of miR-518a-5p were from RiboBio (China). The dual-luciferase reporter gene plasmids were purchased from Shanghai Genechem Co., Ltd. (China). All the plasmids used were identified by DNA sequencing.

### qRT-PCR analysis

Total RNA was extracted from cells with TRIzol reagent (Invitrogen, USA). Nuclear and cytoplasmic RNA were isolated from cells using RNA Subcellular Isolation Kit (Active Motif, USA). Reverse transcription to cDNA was performed using a High Capacity cDNA Reverse Transcription Kit (Thermo Fisher, USA). qRT-PCR analysis was performed using SYBR Green qPCR SuperMix Kit (Vazyme Biotech, China) according to the manufacturer’s protocol. The primer sequences were listed in Supplementary Table 1.

### Western blotting

Total protein was extracted using a protein extraction kit (Vazyme Biotech, China). Equal amounts of protein were separated by 10% SDS–PAGE and then transferred onto PVDF membranes (Millipore). The membranes were blocked with 5% bovine serum albumin (BSA) for 1 h before being incubated overnight at 4°C with the primary antibodies. Then the membranes were washed in TBST and incubated with secondary antibody in 3% BSA-TBST at RT for one hour and washed in TBST. Chemiluminescent detection was performed with Chemi-glow detection reagents (Vazyme Biotech, China). The primary antibodies and the secondary antibody are presented in Supplementary Table 1.

### Cell genomic DNA (gDNA) extraction and electrophoresis

Total gDNA was extracted with a commercial gDNA extraction kit (QIAamp Fast DNA Stool Mini Kit, QIAGEN, #51604) according to the manufacture’s instruction. After successful gDNA extraction, agarose gel electrophoresis was performed in accordance with procedures described in the literature [20].

### RNA FISH assay

RNA FISH assay was performed with the Ribo FISH Kit (RiboBio, China) according to the manufacturer’s instructions. The specific procedure was performed in accordance with the manual provided with the kit. Cells were placed on confocal dishes and incubated at 37°C overnight. Cells were then washed and fixed with 4% paraformaldehyde. Paraformaldehyde-fixed cells were washed with PBS and blocked with blocking solution. Cells were hybridized with probes at 37°C overnight. The cells were then observed and photographed under a confocal fluorescence microscope. For the tumor tissue collected, RNA FISH probe labeling and RNA FISH procedures were performed as described previously [21].

### Transwell assay

Transwell chambers (pore size of 8.0 μm) (Corning) were used to evaluate cell migration in the Transwell assays. Transwell chamber was prepared, 500 μl serum-free DMEM medium was added into the upper chamber, and 5 × 10^5^ cells were seeded in each well. After 24 h of incubation, non-migrated cells were removed with a cotton swab from the upper part of the Transwell and inserts were fixed with 4% paraformaldehyde for 10 min at room temperature. Transwell inserts were stained in 500 μl of 0.1% crystal violet solution. 3 pictures per insert were taken and analyzed.

### Matrigel tube formation assay

Matrigel glue was purchased from Corning company. A Matrigel tube formation assay was performed as previously described. In brief, HUVECs were transfected with miRNA oligonucleotides or plasmid for 24 h; HUVECs were then harvested. 1 × 10^5^ cells were plated on plastic cell-culture dishes coated with Matrigel, and maintained in an incubator at 37°C in 5% CO_2_. The cells were subsequently observed under a light microscope.

### Dual-luciferase reporter gene assay

The dual-luciferase reporter assay was performed in accordance with the manual provided with the Dual Luciferase Reporter Assay Kit (Vazyme, #DD1205-01). Transfection was performed after the amount of plasmid required was calculated and cell lysis and fluorescence intensity assessments were performed 48 h after transfection.

### Animal experiments

All animal studies were conducted with approval from the Animal Research Ethics Committee. Male BALB/c athymic nude mice (4–6 weeks of age, 18–20 g) were provided by the GemPharmatech (China), which were randomly assigned to experimental groups. A total of 2 × 10^5^ cells were injected through the tail vein. Mice were observed daily for weight change for 14 days. Subsequently, the mice were sacrificed using the cervical dislocation method. The liver and lungs were rapidly removed with dissecting instruments and photographed. The removed tissues were fixed in 4% paraformaldehyde as soon as possible for subsequent haematoxylin and eosin (HE) staining.

### Statistical analysis

GraphPad Prism 8.0 was used for data processing. The data were presented as mean ± s.d. or ± s.e.m. Two-sided Student’s *t*-test was used to calculate *P*-values. Statistical significance is displayed as ^*^P < 0.05.

### Data availability statement

The original contributions presented in the study are included in the Article/Supplementary Material. Further inquiries can be directed to the corresponding authors.

## RESULTS

### Screening of angiogenesis-related circRNAs in CRC

**Figure 1:**
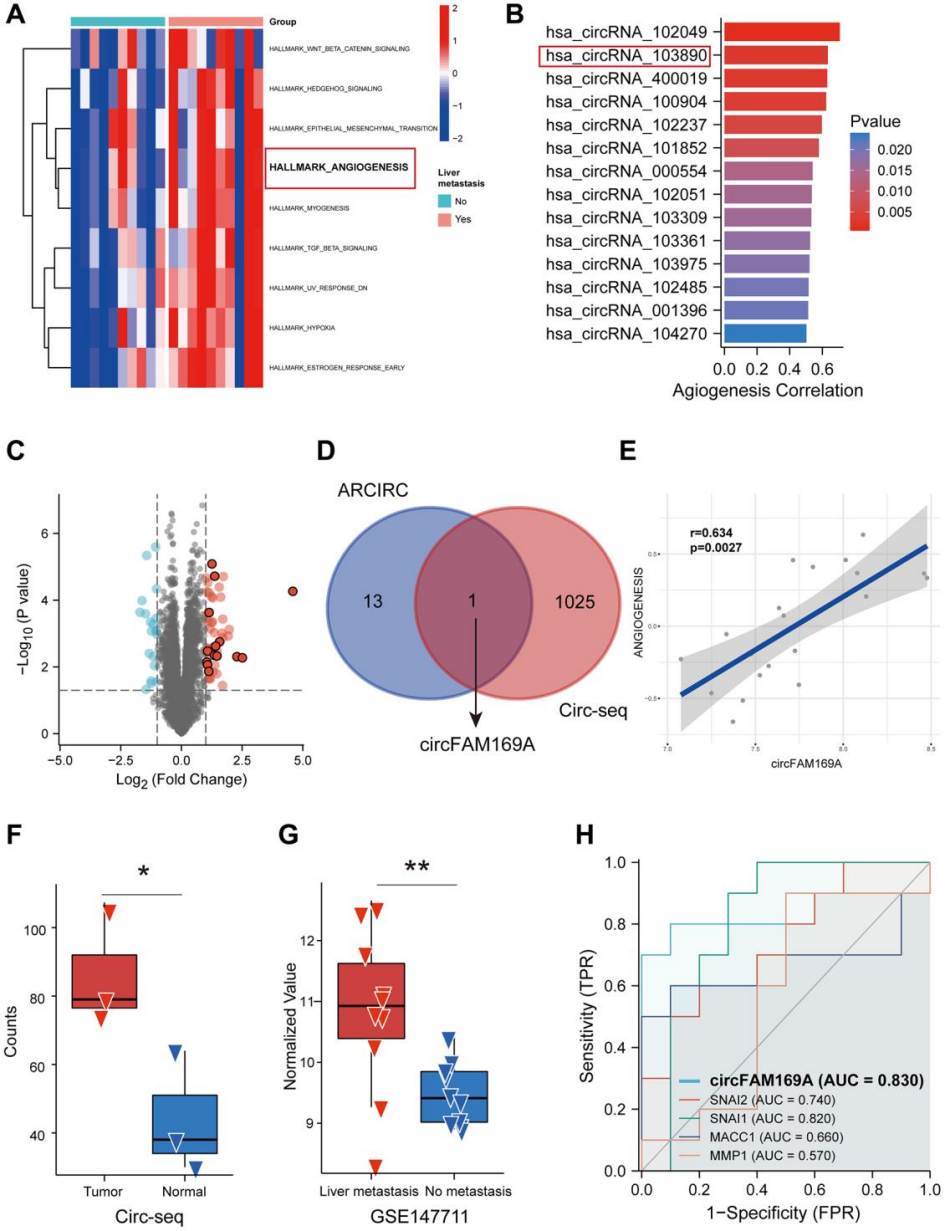
**Screening of angiogenesis-related circular RNAs (circRNAs) in colorectal cancer (CRC).** (**A**) Heatmap showing the up-regulated hallmarks in CRC patients with liver metastasis. (**B**) Spearman’s correlation analysis showing the correlation between circRNA expression and angiogenesis scores assessed by gene set variation analysis (GSVA). (**C**) Volcano diagram of circRNAs differentially expressed between CRC patients with and without liver metastasis. (**D**) Venn diagram of circRNAs highly associated with angiogenesis and upregulated circRNAs. (**E**) Correlation analysis between circFAM169A and angiogenesis scores assessed by GSVA. (**F**) Expression level of circFAM169A in CRC samples and non-tumoral tissues from GSE13572. (**G**) Expression level of circFAM169A in CRC patients with and without liver metastasis from GSE147711. (**H**) Receiver operating characteristic (ROC) curve showing the diagnostic value of circFAM169A compared with that of SNAI1, SNAI2, MACC1, and MMP1.

CRC patient data from an online dataset (GSE147711) were subjected to GSVA to compare the differential expression of hallmark signatures between patients with and without liver metastasis. The hallmark of angiogenesis was significantly overexpressed in CRC patients with liver metastases (Figure 1A), corroborating the significant role angiogenesis plays in CRC progression. CircRNAs that were highly correlated with angiogenesis were identified using Spearman’s correlation analysis (Figure 1B). Then, we identified 1026 up-regulated circRNAs in CRC samples between normal and tumour tissue based on our own circRNA sequence data (Figure 1C). Subsequently, we focused on circRNA_103890 (circFAM169A) (Figure 1D), which was highly correlated with angiogenesis (Figure 1E, r = 0.634) and highly expressed in CRC tissues compared with normal tissues (Figure 1F). Meanwhile, its expression was higher in liver-metastasized CRC compared with primary CRC (Figure 1G). The receiver operating characteristic curve showed that circFAM169A had a higher diagnostic value (area under the curve = 0.83) than some widely studied metastasis-related mRNAs, such as SNAI1, SNAI2, MACC1, and MMP1 (Figure 1H). Therefore, circFAM169A was proved to be important in CRC angiogenesis. This is also the first instance of validating the high correlation between angiogenesis and CRC metastasis using creative bioinformatic analyses.

### Identification of the potential biological functions of circFAM169A in CRC

**Figure 2:**
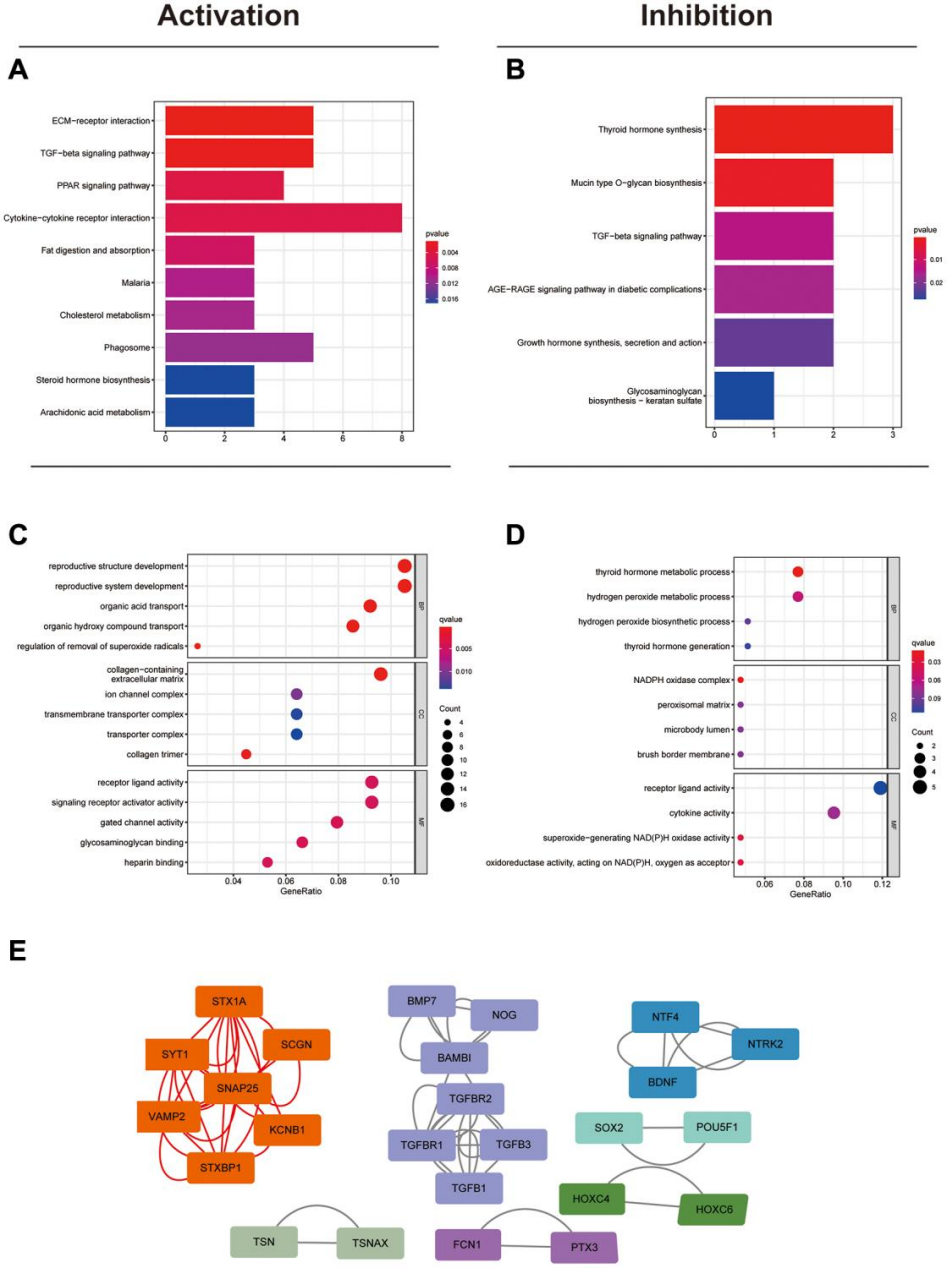
**Identification of the potential biological functions of circFAM169A in CRC.** (**A**) Kyoto Encyclopedia of Genes and Genomes (KEGG) analysis showed that many metastasis-related pathways were activated. (**B**) KEGG analysis showed that some synthesis-related pathways were inhibited. (**C**) Gene Ontology (GO) analysis showing the biological processes that were activated. (**D**) GO analysis showing the biological processes that were inhibited. (**E**) Protein–protein interaction network showing the possible protein networks that might be regulated by circFAM169A.

To elucidate the possible roles of circFAM169A in CRC angiogenesis and metastasis, we analyzed the differences between the five groups with the highest and lowest circFAM169A expression, and screened out the candidate mRNAs that may be up- or down-regulated by circFAM169A. The identified gene clusters were analyzed using the Kyoto Encyclopedia of Genes and Genomes to discover stimulatory or inhibitory pathways (Figure 2A, 2B, Supplementary Table 2). Two pathways known to be involved in tumor metastasis – extracellular matrix receptor interaction pathways and transforming growth factor-beta signaling – were highly correlated with circFAM169A expression. In addition, Gene Ontology analysis implicated circFAM169A in BB, MF, and CC (Figure 2C, 2D). The protein–protein interaction network showed that circFAM169A may affect the interaction network of related proteins (Figure 2E).

### Characteristics of circFAM169A in CRC

**Figure 3:**
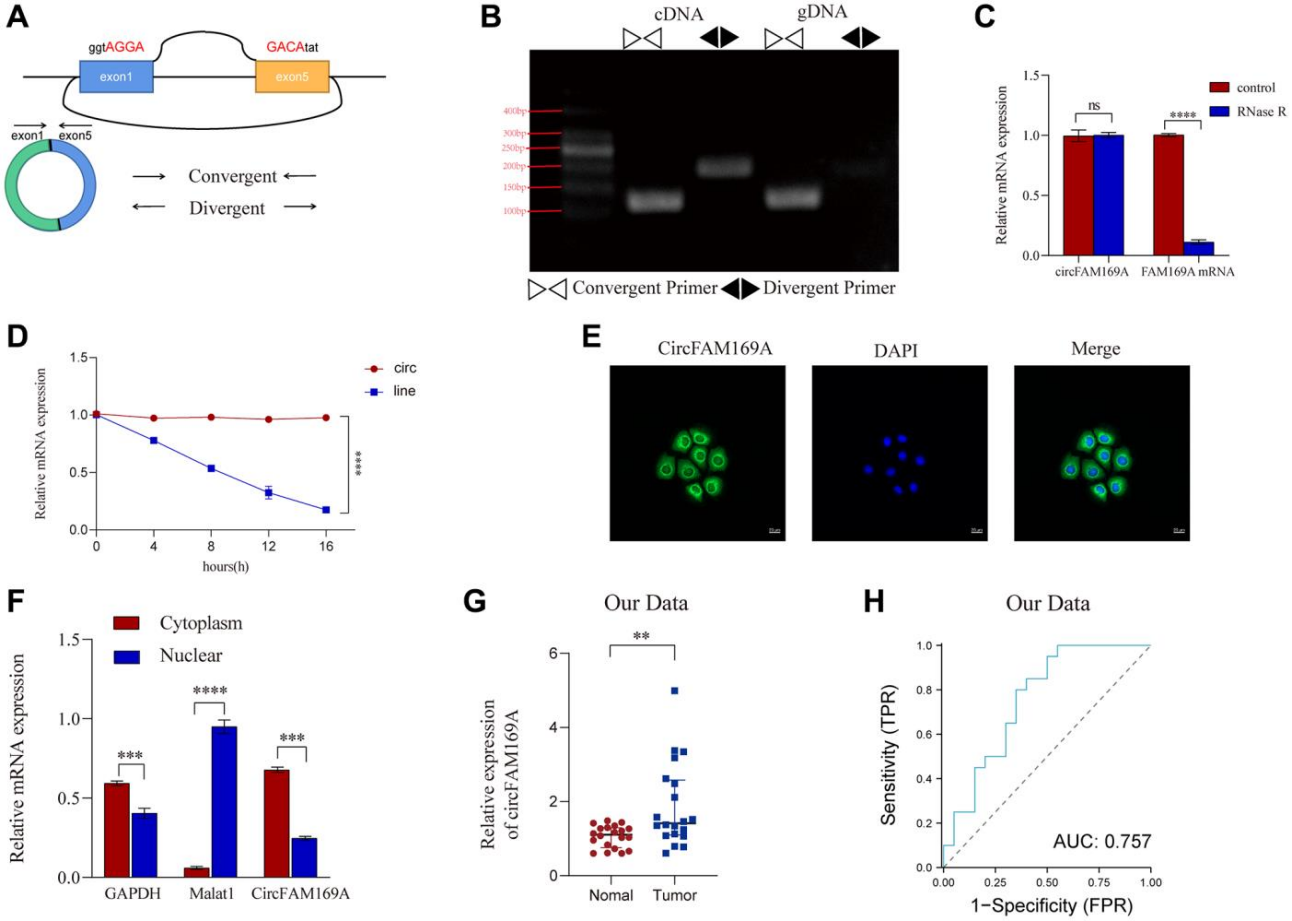
**Characteristics of circFAM169A in CRC cells.** (**A**) The diagram represents the structure of circFAM169A and the arrows denote the different primers. (**B**) The reverse transcription–PCR (RT-PCR) products from different primers were detected by agarose gel electrophoresis. (**C**) Samples were treated with RNase (+) or buffer (−), and the expression of circFAM169A was determined by quantitative RT–PCR (qRT–PCR). (**D**) SW480 cells were treated with (+) or without (−) actinomycin D, and the stability of circFAM169A was assessed by qRT–PCR. (**E**) Cells were analyzed by immunofluorescence cytochemistry. Scale bar = 20 μm. (**F**) The location of circFAM169A, GAPDH, and Malat1 in CRC cells. (**G**) Relative expression of circFAM169A in 20 paired CRC tumor and adjacent normal tissues determined using qRT–PCR. (**H**) ROC curves illustrating the capacity of circFAM169A to distinguish between tumors and adjacent tissues from patients with CRC. All data are presented as means ± standard deviation (SD) (*n* = 3 independent experiments). ^*^p ≤ 0.05, ^**^p ≤ 0.01, ^***^p ≤ 0.001, and ^****^p ≤ 0.0001.

A pair of convergent and divergent primers was designed to target the loop-forming sites of circFAM169A (Figure 3A). Agarose gel electrophoresis revealed that the convergent primer could show bands in both cDNA and gDNA, whereas the divergent primer only showed bands in cDNA (Figure 3B). Compared with FAM169A mRNA, circFAM169A resisted RNase R digestion and was degraded much slower by actinomycin, indicating its greater stability (Figure 3C, 3D). circFAM169A was mainly localized in the cytoplasm of CRC cells, as evidenced by fluorescence *in situ* hybridization assays using clinical CRC tissues and CRC cells (Figure 3E) and nucleus–cytoplasm separation assay (Figure 3F). The relative expression of circFAM169A in 20 pairs of carcinoma and paracancerous samples was measured by quantitative reverse transcription–PCR. circFAM169A was found highly expressed in CRC tissues (Figure 3G). The receiver operating characteristic curve also demonstrated that the expression level of circFAM169A can effectively distinguish between carcinoma and paracancerous tissues (Figure 3H). Furthermore, a correlation analysis showed that circFAM169A was associated with lymphatic CRC metastasis (pN stage) (Table 1). These experiments further highlight the potential involvement of circFAM169A in tumor metastasis.

**Table 1:** Clinic-pathological characteristics of enrolled patients.

**Clinical parameters**	**Total**	**circFAM169A**		***p* Value**
		**High (%)**	**Low (%)**	
**Gender**				
Female	11	5 (45.4)	6 (54.6)	0.653
Male	9	5 (55.6)	4 (44.4)	
**Age (years)**				
≤60	4	2 (50.0)	2 (50.0)	1
>60	16	8 (50.0)	8 (50.0)	
**Pathologic stage**				
Stage I–II	9	5 (55.5)	4 (45.5)	0.653
Stage III–IV	11	5 (45.5)	6 (54.5)	
**Pathology T stage**				
T1–T2	8	4 (50.0)	4 (50.0)	1
T3–T4	12	6 (50.0)	6 (50.0)	
**Pathology *N* stage**				
N0	0	0 (0)	0 (0)	0.05
N1	14	5 (35.7)	9 (64.3)	
N2	6	5 (83.3)	1 (16.7)	
**Pathology M Stage**				
M0	18	9 (50.0)	9 (50.0)	1
M1	2	1 (50.0)	1 (50.0)	
**Tumor size**				
≥5 cm	10	6 (60.0)	4 (60.0)	0.371
<5 cm	10	4 (40.0)	6 (40.0)	
**Tumor site**				
Left Colon	1	0 (0)	1 (100.0)	0.564
Right Colon	12	6 (50.0)	6 (50.0)	
Rectum	7	4 (57.1)	3 (42.9)	

### circFAM169A promotes CRC angiogenesis and metastasis *in vitro* and *in vivo*

**Figure 4:**
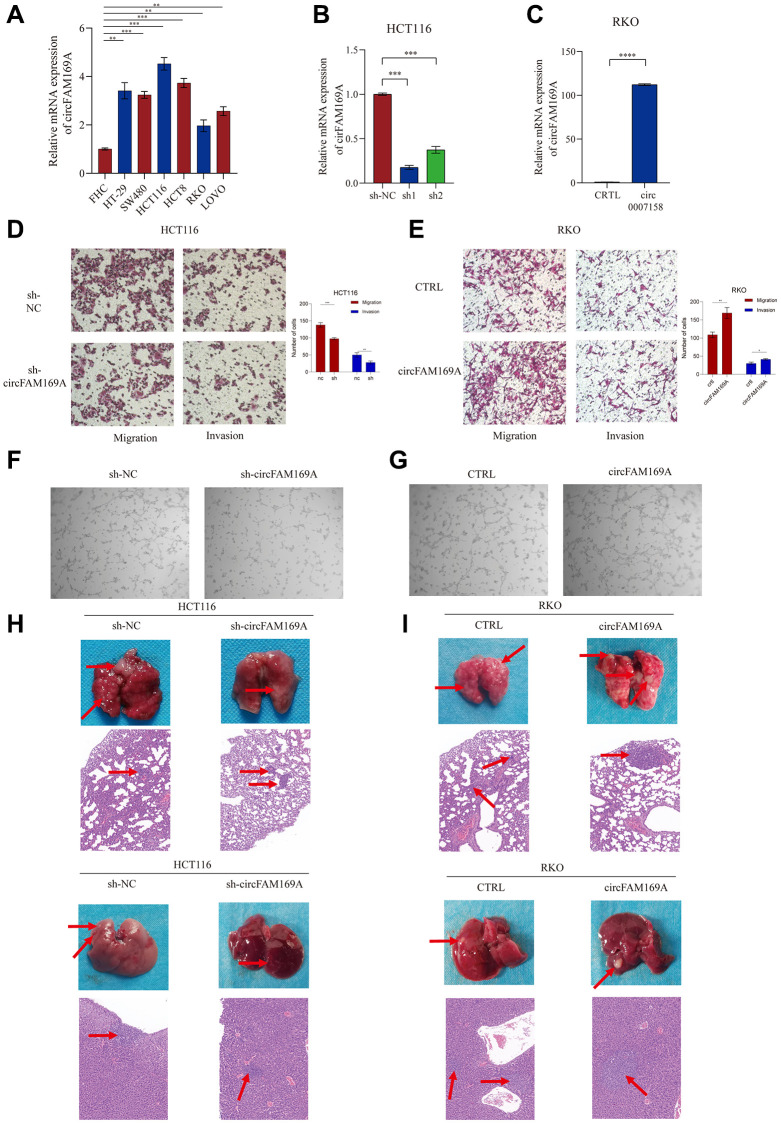
**circFAM169A promotes CRC angiogenesis and metastasis *in vitro* and *in vivo*.** (**A**) The expression of circFAM169A in FHC, HT29, SW480, HCT116, HCT8, LOVO, and RKO cells determined using qRT–PCR. All data are presented as means ± SD (*n* = 3 independent experiments). ^*^p ≤ 0.05, ^**^p ≤ 0.01, ^***^p ≤ 0.001, and ^****^p ≤ 0.0001. (**B**, **C**) Construction of stable inducible circFAM169A-knockdown or -overexpressed CRC cell lines. All data are presented as means ± SD (*n* = 3 independent experiments). ^***^p ≤ 0.001 and ^****^p ≤ 0.0001. (**D**, **E**) Transwell migration assay and Transwell invasion assay were performed using HCT116 and RKO cells. (**F**, **G**) An *in vitro* Matrigel tube formation assay was performed to evaluate the angiogenic ability of human umbilical vein endothelial cells (HUVECs). The representative micrographs are shown at 200× magnification. (**H**, **I**) Representative images and bar graphs of liver and lung metastases with circFAM169A-knocked down HCT116 cells (**H**) and circFAM169A-overexpressing SW480 cells (**I**) in a nude mouse metastatic tumor model.

The expression of circFAM169A was significantly upregulated in the CRC cell lines HCT116, HT29, HCT8, LOVO, RKO, and SW480 compared with that in the epithelial cell line FHC. It was especially high in HCT116, and relatively lower in RKO (Figure 4A), so we selected these two cell lines for functional experiments. circFAM169A was stably knocked down using short hairpin RNA in HCT116 cells (Figure 4B); shcircFAM169A#1 was employed in subsequent assays. Conversely, circFAM169A was stably up-regulated in RKO cells using a circFAM169A vector (Figure 4C). Transwell assay demonstrated that circFAM169A knockdown attenuated the migratory and invasive potential of HCT116 cells (Figure 4D), while circFAM169A up-regulation boosted these abilities in RKO cells (Figure 4E). Next, we investigated whether circFAM169A could regulate angiogenesis *in vitro* using the tube formation assay. Compared with the control, tumor-conditioned medium (TCM) from circFAM169A-knocked down HCT116 cells inhibited human umbilical vein endothelial cell tube formation (Figure 4F), whereas TCM from circFAM169A-overexpressing RKO cells induced tube formation (Figure 4G), further corroborating that circFAM169A could regulate angiogenesis *in vitro*. Subsequently, we constructed an HCT116 and RKO nude mouse metastatic tumor model. Compared with the control, a lower number of liver and lung metastatic nodules were observed in mice wherein circFAM169A had been down-regulated (Figure 4H), while increased numbers of liver and lung metastatic nodules were observed when circFAM169A was overexpressed (Figure 4I).

### circFAM169A promotes CRC by sponging miR-518a-5p

**Figure 5:**
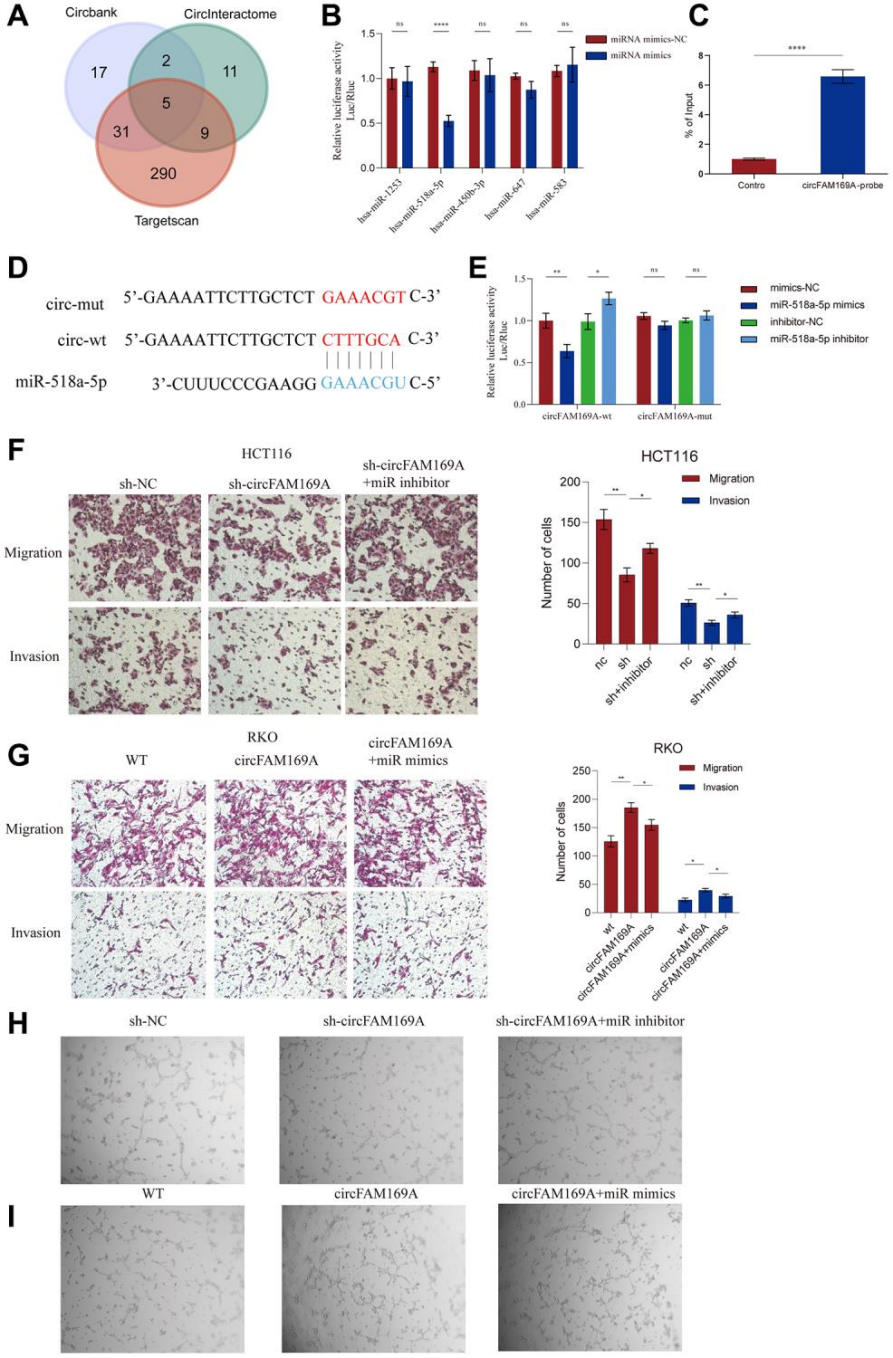
**circFAM169A promotes CRC by sponging miR-518a-5p.** (**A**) Venn diagram of miRNA response elements in circBank, CircInteractome, and TargetScan that may interact with circFAM169A. (**B**) The luciferase activities of different plasmids were measured by a dual-luciferase assay. (**C**) RNA antisense purification (RAP) assay showed that miR-518a-5p was significantly enriched when circFAM169A was used as a probe. (**D**) Construction of a luciferase plasmid encoding mut-circFAM169A. (**E**) The luciferase activities of different groups were measured by a dual-luciferase assay. (**F**, **G**) Transwell migration assay and Transwell invasion assay were performed using HCT116 and RKO cells. (**H**, **I**) An *in vitro* Matrigel tube formation assay was performed to evaluate the angiogenic ability of HUVECs. The representative micrographs are shown at 200× magnification.

Since most circRNAs typically function as ceRNAs, to further understand the mechanism of circFAM169A in CRC, we explored the miRNA response elements that might interact with circFAM169A using online databases, such as circBank, CircInteractome, and TargetScan. We obtained five candidates: miR-1253, miR-518A-5p, miR-450b-3p, miR-647 and miR-583 (Figure 5A, Supplementary Table 3, Supplementary Figure 1A). To characterize their interaction with circFAM169A, a circFAM169A-encoding luciferase plasmid was co-transfected with each of the five miRNA mimics in a Renilla luciferase plasmid. The fluorescence intensity of miR-518a-5p significantly dropped after co-transfection, suggesting that these two RNA moieties interact (Figure 5B) and miR-518a-5p significantly down-regulated in CRC samples compared with normal tissues (Supplementary Figure 1B). RNA antisense purification assay showed that miR-518a-5p was significantly enriched when circFAM169A was used as a probe (Figure 5C). We also constructed a luciferase plasmid encoding mut-circFAM169A (Figure 5D). The dual-luciferase reporter assay showed that after transfecting cells with circFAM169A, miR-518a-5p mimics significantly reduced the fluorescence intensity while miR-518a-5p inhibitor increased it. When mut-circFAM169A was used, the fluorescence intensity was not altered significantly, implying that miR-518a-5p interacts with a specific region of circFAM169A (Figure 5E). Moreover, the impaired migration and invasion of circFAM169A-knocked down HCT116 cells were rescued by miR-518a-5p mimics (Figure 5F), while the enhanced migration and invasion of circFAM169A-overexpressing RKO cells were reversed by miR-518a-5p inhibitor (Figure 5G, Supplementary Figure 1C). Importantly, tube formation assays showed that miR-518a-5p mimics or inhibitors could successfully rescue the effects of TCM derived from circFAM169A-knocked down HCT116 cells or circFAM169A-overexpressing RKO cells (Figure 5H, 5I).

### circFAM169A enhances CRC angiogenesis and metastasis by targeting ANGPT2

**Figure 6:**
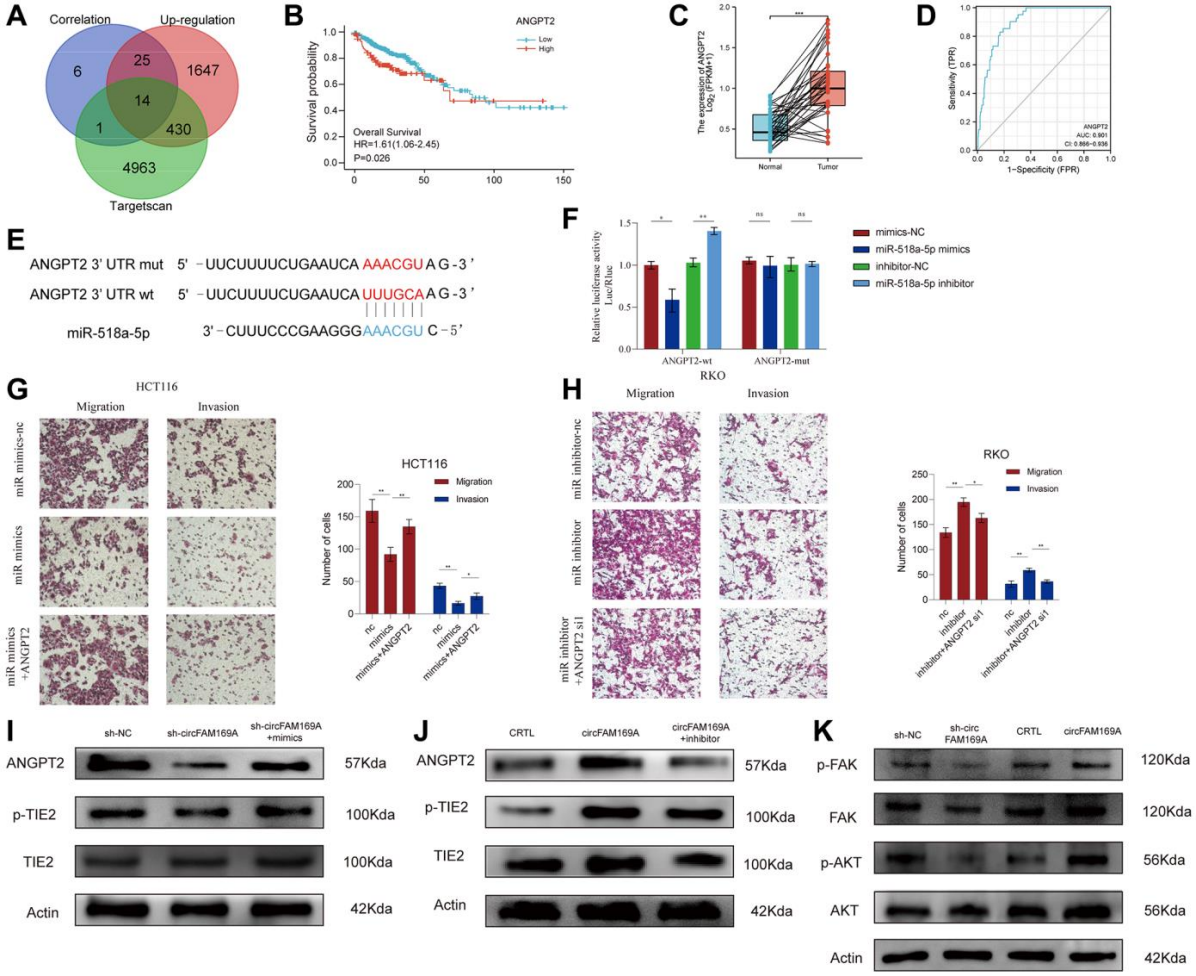
**circFAM169A enhances CRC angiogenesis and metastasis by targeting angiopoietin-2 (*ANGPT2*).** (**A**) Venn diagram of mRNA that was highly correlated with circFAM169A and the downstream target genes of miR-518a-5p as predicted by TargetScan. (**B**) Survival analysis of ANGPT2. (**C**) ANGPT2 expression in normal and tumor tissues. The luciferase activities were measured by a dual-luciferase assay, and the Renilla/firefly luciferase light-unit ratio was measured. (**D**) ROC curve showed the diagnostic value of ANGPT2. (**E**) Construction of a luciferase plasmid encoding mut-ANGPT2. (**F**) The luciferase activities of different groups were measured by a dual-luciferase assay. (**G**, **H**) Transwell migration assay and Transwell invasion assay were performed using HCT116 and RKO cells. All data are presented as means ± SD (*n* = 3 independent experiments). ^***^p ≤ 0.001 and ^****^p ≤ 0.0001. (**I**) Western blotting analysis of ANGPT2, total TIE2, and p-TIE2 expression in circFAM169A-knocked down HCT116 cells treated with miR-518a-5p mimics. (**J**) Western blotting analysis of ANGPT2, total TIE2, and p-TIE2 expression in circFAM169A-overexpressing RKO cells treated with miR-518a-5p inhibitors. (**K**) Western blotting analyses of total FAK, p-FAK, total AKT, and p-AKT in CRC cells with different circFAM169A expression levels.

To determine the potential targets of circFAM169A, we used TargetScan to identify 5408 genes that might interact with it. From these, genes that were up-regulated (fold change >2) and highly correlated with circFAM169A expression (r > 0.7) were selected to yield 14 potential targets of the circFAM169A/miR-518a-5p axis, including ANGPT2 (Figure 6A, Supplementary Table 4).

Using CRC data from The Cancer Genome Atlas (TCGA) database, we found that ANGPT2 was correlated with poor overall survival rate (*P* = 0.026, Figure 6B), and significantly up-regulated in CRC samples compared with normal tissues (Figure 6C). ROC curves further demonstrated that the expression level of ANGPT2 can effectively distinguish between carcinoma and paracancerous tissues (Figure 6D). After transfecting cells with a luciferase plasmid encoding the ANGPT2 3′-untranslated region, miR-518a-5p mimics significantly reduced the fluorescence intensity while miR-518a-5p inhibitor increased it. Importantly, the fluorescence intensity was unchanged when the transfected luciferase plasmid encoded a mutant ANGPT2 sequence, indicating that ANGPT2 and miR-518a-5p interact with each other (Figure 6E, 6F). Like circFAM169A, the expression of *ANGPT2* was higher in the cancerous HCT116, HT29, RKO, and SW480 cell lines than in the normal epithelial FHC cells (Supplementary Figure 1D). Moreover, *ANGPT2* overexpression successfully rescued the miR-518a-5p mimic-induced impaired invasion and migration of HCT116 cells (Figure 6G, Supplementary Figure 1E). Meanwhile, small interfering *ANGPT2* reduced the miR-518a-5p inhibitor-induced invasion and migration of RKO cells (Figure 6H, Supplementary Figure 1F). These data suggest that ANGPT2 is the direct target of miR-518a-5p in promoting CRC metastasis.

ANGPT2 can competitively bind to Tie2, thereby interfering with the ANGPT1/Tie2 pathway and inhibiting the phosphorylation of Tie2 [22, 23]. The focal adhesion kinase (FAK)/AKT signaling pathway plays a fundamental role in angiogenesis [24, 25]. Using Western blotting, we showed that circFAM169A knockdown increased Tie2 phosphorylation, and this trend was partially reversed by miR-518a-5p mimics (Figure 6I). Conversely, when circFAM169A was overexpressed, p-Tie2 significantly decreased, and could be partially rescued by miR-5p inhibitor (Figure 6J). More importantly, shcircFAM169A treatment reduced the levels of p-FAK and p-AKT while the overexpression of circFAM169A increased them (Figure 6K). Overall, these results prove that the circFAM169A/miR-518a-5p/ANGPT2 axis is essential in promoting CRC angiogenesis.

## DISCUSSION

CircRNAs are newly defined non-coding RNAs that play an important role in the growth and metastasis of many cancers, attracting many investigators to explore their functions and mechanism [26, 27]. In this study, we first identified that circFAM169A is highly correlated with angiogenesis through bioinformatic analyses, and that the circFAM169A/miR-518a-5p/ANGPT2 axis can regulate the angiogenesis and metastasis of CRC.

Angiogenesis has long been considered an important cause of tumor metastasis [28, 29]. Some circRNAs can regulate cancer angiogenesis [8, 30], but studies on pro-angiogenic circRNAs are limited and a method effectively targeting angiogenesis-related circRNAs is lacking.

In this study, we directly screened specific circRNAs that were highly correlated with angiogenesis using GSVA, specifically focusing on circFAM169A, which was identified using circRNA microarray data from CRC patients. *In vitro* and *in vivo* experiments further validated the ability of circFAM169A to promote angiogenesis and CRC metastasis. In addition, circFAM169A was highly expressed in tissues from CRC patients, suggesting its potential as a diagnostic marker for CRC.

Many studies have proposed that circRNAs function as a part of the ceRNA network: they regulate mRNA expression post-transcriptionally by competing for shared miRNAs [31]. In this study, we found that circFAM169A is mainly located in the nucleus. Bioinformatic and dual-luciferase reporter analyses showed that circFAM169A competitively adsorbed miR-518a-5p, inhibiting its effect on the downstream gene ANGPT2, thereby promoting CRC angiogenesis and metastasis. ANGPT2 is a key functional gene influencing angiogenesis. Conditions like neovascular age-related macular degeneration and diabetic macular edema could be successfully treated by inhibiting ANGPT2 [32]. Conversely, ANGPT2 has also been shown to promote angiogenesis in cancers [33]. No study has ever reported that ANGPT2 can be modulated by a circRNA. For the first time here, we identified that ANGPT2 can be up-regulated by circFAM169A. It promotes angiogenesis by competitively binding to Tie-2, ultimately supporting the metastasis of CRC. Furthermore, Angpt2 has been reported to trigger the phosphorylation of focal adhesion kinase (FAK) signaling pathways [34]. FAK is a non-receptor tyrosine kinase and an adaptor protein that mediates adhesion signaling and cell migration. FAK is frequently overexpressed in cancers and is regarded as a valuable target for cancer therapy [35, 36]. A recent study has shown that FAK phosphorylation in endothelial cells is indispensable for angiogenesis. It plays a pivotal role in vascular remodeling by modulating Tie2, VEGFR2 and β1 integrin [37] and can also promote angiogenesis by inducing vasculogenic mimicry (VM), which is a process where tumor cells form vessel-like structures that can transport blood independently of endothelial cells [38]. Moreover, FAK phosphorylation rapidly activates AKT signaling pathway [39]. In this study, we demonstrated for the first time that circFAM169A upregulates ANGPT2, which activates the phosphorylation of FAK and AKT, and thereby enhances angiogenesis.

## CONCLUSION

Using GSVA, circRNA microarray data, and *in vitro* and *in vivo* experiments, we found for the first time that circFAM169A is highly expressed in the tissues of CRC patients and is highly correlated with angiogenesis. circFAM169A promotes CRC angiogenesis via the miR-518a-5p/ANGPT2 axis. Despite uncovering the specific function and mechanism of circFAM169A in CRC, this study still has some limitations. The CRC sample size in this study was still too small to identify the diagnostic value of circFAM169A. The potential of circFAM169A as a therapeutic target also needs to be further investigated.

## Supplementary Materials

Supplementary Figure 1

Supplementary Table 1

Supplementary Table 2

Supplementary Table 3

Supplementary Table 4
