# Supplementary material for: The novel angiogenesis regulator circFAM169A promotes the metastasis of colorectal cancer through the angiopoietin-2 signaling axis
Source: Aging (Albany NY). 2023 Aug 23;15(16):8367–83. doi: 10.18632/aging.204974 (PMC10496999; doi:10.18632/aging.204974)
Supplement: Supplementary Figure 1 [file aging-15-204974-s001.pdf]

SUPPLEMENTARY FIGURE

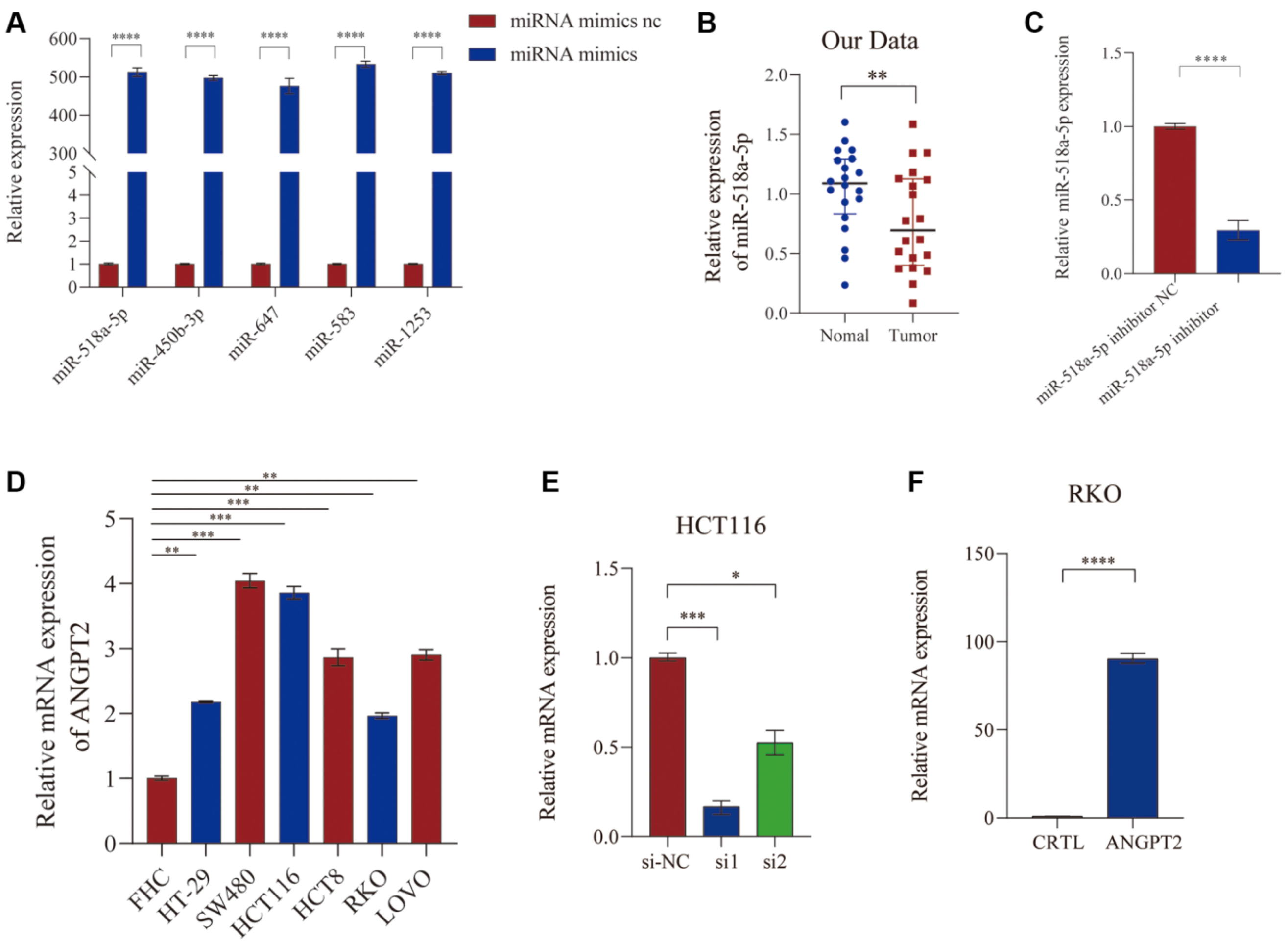

Supplementary Figure 1. (**A**) qRT-PCR detection of the transfection efficiency of miRNA mimics. (**B**) qRT-PCR detection of the miR-518a-5p expression levels in tumors and adjacent tissues from patients with CRC. (**C**) qRT-PCR detection of the transfection efficiency of the miR-342-5p inhibitor. (**D**) The expression of ANGPT2 in FHC, HT29, SW480, HCT116, HCT8, LOVO, and RKO cells determined using qRT–PCR. (**E**, **F**) Construction of stable inducible ANGPT2-knockdown or -overexpressed CRC cell lines. All data are presented as means ± SD (*n* = 3 independent experiments). $^{*}p \leq 0.05$, $^{**}p \leq 0.01$, $^{***}p \leq 0.001$ and $^{****}p \leq 0.0001$.
